# Epidemiological trends and outcomes of extensively drug-resistant tuberculosis in Shandong, China

**DOI:** 10.1186/s12879-017-2652-x

**Published:** 2017-08-09

**Authors:** Xiao-chun He, Ning-ning Tao, Yao Liu, Xian-xin Zhang, Huai-chen Li

**Affiliations:** 1 Department of Respiratory Medicine, Baoji Central Hospital, Baoji, Shaanxi China; 2 Department of Respiratory Medicine, Shandong Provincial Hospital Affiliated to Shandong University, Jinan, Shandong China; 3 Department of Respiratory Medicine, Shandong Provincial Chest Hospital, Jinan, Shandong China

**Keywords:** XDR-TB, Epidemiology trends, Treatment outcomes, Survival

## Abstract

**Background:**

Extensively Drug-Resistant (XDR) Tuberculosis (TB) has posed a great threat to global health and finance systems, especially for developing countries with high TB and Multidrug-Resistant (MDR) TB burden.

**Methods:**

We retrospectively analyzed HIV-uninfected TB case confirmed and treated in Shandong Provincial Chest Hospital (SPCH) between January 2008 and December 2015. Unique characteristics of XDR-TB were identified; its longitudinal changes and survival were analyzed.

**Results:**

Between January 2008 and December 2015, a total of 144 cases were confirmed to be XDR-TB (2.5% of 5663 culture-confirmed TB cases; 27.9% of 516 MDR-TB cases). The proportion of XDR TB cases among MDR-TB cases has increased from 26.5% in 2008 to 44.5% in 2014 (Chi-Square test for trends: *P* < 0.01). Among the 144 XDR-TB cases, 21 patients (14.6%) had treatment success, 123 (85.1%) had poor treatment outcomes. Mortality was higher among XDR-TB cases than among MDR TB cases (8.3% vs. 3.8%, *P* = 0.033) and drug-susceptible TB cases (8.3% vs. 2.1%, *P* < 0.01).

**Conclusions:**

XDR-TB cases comprise a substantial and increasing fraction of MDR-TB cases, causing poor treatment outcomes and high mortalities. Early drug susceptibility testing, adequate TB treatment and efficient infection control must be in place in future TB control strategies.

## Background

Extensively drug-resistant tuberculosis (XDR-TB), which is defined as tuberculosis resistant to isoniazid, rifampin, a fluoroquinolone, and a second-line injectable agent, has posed a great threat to global public health [1–3]. XDR TB is a subset of multidrug-resistant (MDR) TB, which is resistant to both isoniazid and rifampin [4]. Rooted in inappropriate TB treatment [5–8] and compounded by the neglect of airborne infection control [9, 10], the emergence and spread of XDR-TB worldwide reflect the weakness in global TB management. XDR-TB puts TB patients in a predicament with limited therapeutic options that are more toxic, more costly, and less effective and consequently have poorer treatment outcomes and higher mortality [11–14].

In 2014, an estimated 650,000 (5.4%) of the world’s 12 million prevalent cases of TB had MDR-TB. According to WHO, about 9.7% of people with MDR-TB have XDR-TB. The proportion of XDR-TB among MDR-TB cases was highest in Belarus (29%), Georgia (15%), Latvia (19%) and Lithuania (25%) [15]. China is a country of high TB and MDR-TB burden and a countrywide survey in 2007 revealed that the proportion of XDR-TB among MDR-TB cases was 8% [15, 16]. Nevertheless, XDR TB is probably an underestimated problem in mainland China; recent studies [17–19] suggested that the proportion of XDR-TB may be substantially higher than previously reported. However, systematic data on the epidemiology of XDR-TB remain limited, and most reports focus on populations from special surveys or go through short time intervals, information such as longitudinal changes and survival analysis has not yet been reported in mainland China.

This study was based on clinical information collected from the only provincial TB specialized hospital in Shandong province. We aimed to determine XDR-TB burden more accurately by considering all reported drug susceptibility test results for case counting and by analyzing secular trends across an 8-year study period. To identify specific characteristics of XDR-TB, we compared the basic characteristics and survival of XDR-TB cases with both MDR-TB and drug-susceptible TB cases.

## Methods

### Ethics statement

The study was approved by the Ethic Committee of Shandong Provincial Hospital, affiliated to Shandong University. Patient records were anonymized and de-identified prior to analysis.

### Study population and data collection

Shandong province lies in the east coast of China and is the second largest province with a population of 95 million. In 2013, 35,971 people in Shandong were infected with latent TB. Shandong Provincial Chest Hospital (SPCH) is the only provincial-level hospital specializing in TB clinical service and control. This retrospective study enrolled consecutive human immunodeficiency virus (HIV)-uninfected culture-positive Mycobacterium tuberculosis cases confirmed and treated in SPCH between January 2008 and December 2015. Trained research clinicians reviewed the medical records of these TB patients and abstracted their drug susceptibility test (DST) results along with the corresponding sociodemographic and clinical information using a standard case report form.

### Drug susceptibility testing

All the enrolled TB patients had DST results for first line antituberculosis drugs isoniazid, rifampin, ethambutol, streptomycin, and, for at least 1 fluoroquinolone (ofloxacin, levofloxacin, ciprofloxacin) and 1 injectable agent (capreomycin, kanamycin, amikacin).

DST was performed using the proportion method with Löwenstein–Jensen (L-J) medium. The critical concentrations for resistance were as follows [20]: isoniazid (0.2 μg/mL), rifampicin (40 μg/mL), ethambutol (2.0 μg/mL), streptomycin (4.0 μg/mL), ofloxacin (2.0 μg/mL), levofloxacin (1.0 μg/mL), ciprofloxacin (2.0 μg/mL), capreomycin (40 μg/mL), kanamycin (30 μg/mL), amikacin (10 μg/mL) and para-aminosalicylic (1.0 μg/mL). External quality assessment was conducted regularly by TB National Reference Laboratory.

### Definitions

New TB cases were defined as TB patients who had never been treated for TB or had taken anti-TB drugs for less than 1 month. Previously treated TB cases were those who had been receiving TB treatment for at least 1 month. A drug-susceptible TB case was defined as an individual susceptible to isoniazid and rifampin in both initial and follow-up DST results. An MDR-TB case was defined as a person resistant to at least isoniazid and rifampin. MDR-TB cases that had additional resistance to a fluoroquinolone and a second line injectable drug were defined as XDR-TB cases.

Treatment outcomes were defined according to the WHO and International Union Against Tuberculosis and Lung Disease (IUATLD) guidelines [21, 22]. “Cured” was defined as one who had completed treatment and had been consistently culturenegative (with at least 5 results) for the final 12 months of TB treatment. Patients who completed treatment but did not meet the definition for cure because of lack of bacteriological results were considered to have “completed treatment”. Patients who did not receive treatment for ≥ 2 consecutive months were defined as having “defaulted treatment”. “Treatment Failure” was defined as ≥ 2 positive cultures recorded in the final 12 months or if any 1 of the final three cultures was positive. “Transferred out” means anyone who transferred to another institution and for whom the treatment outcome was unknown. “Death” was defined as death during treatment due to any cause. For analysis purposes, “cured” and “completed treatment” were classified as “Treatment Success”, whereas others were classified as “Poor Treatment Outcome”.

### Statistical analysis

To identify unique features of XDR-TB, we compared sociodemographic and clinical characteristics of XDR-TB with MDR-TB and drug-susceptible TB, respectively. Categorical variables were compared using Chi-square Test or, when appropriate, Fisher’s exact test; continuous variables were analyzed with use of the Mann-Whitney U test. Chi-Square test for trends and linear regression were used to assess the changes in proportions of XDR-TB over time. Unadjusted survival rates were estimated by the Kaplan-Meier method, and any differences in survival were evaluated with the log-rank test. Multivariate Cox regression models were used to estimate the simultaneous effects of prognostic factors on survival. Death was the event variable and other outcomes were censored. Tests of significance were 2-sided, and a *P* value <0.05 was considered statistically significant. All analyses were performed by using SPSS software, version 16.0.

## Results

### Drug resistance patterns

**Fig. 1:**
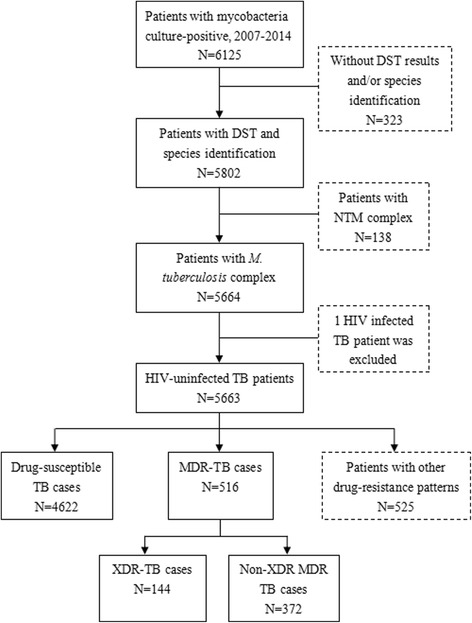
TB cases in Shandong, China. NTM, *nontuberculous mycobacteria*; MDR, multi-drug resistant; XDR, extensively drug resistant

Between January 2008 and December 2015, a total of 5664 culture-confirmed Mycobacterium tuberculosis cases were reported and treated at SPCH. Of these, 1 patient co-infected with HIV was excluded from further analysis. Among the remaining 5663 TB cases, 4622 (81.6%) cases were drug-susceptible. There were 516 (9.1%) cases with resistance to at least isoniazid and rifampin, meeting the case definition for MDR-TB. In our study, all the MDR-TB cases had susceptibilities reported for at least 1 fluoroquinolone and 1 injectable agent, namely, all the MDR-TB cases were XDR-TB evaluable. Ultimately, 144 cases were confirmed to have XDR-TB (2.5% of 5663 culture-confirmed TB cases; 27.9% of 516 MDR-TB cases). We excluded 525 TB cases with other drug resistance patterns from further analysis (Fig. 1).

Treatment histories were available for the whole study cohort. Out of the 5663 TB cases, 4999 (88.3%) were new TB cases and 664 (11.7%) were previously treated TB cases. In new TB cases, the proportions of MDR TB and XDR TB were 4.2% and 1.5%, respectively. While the corresponding proportions were 24.7% and 10.7% in previously treated TB cases.

### Trends over time

**Fig. 2:**
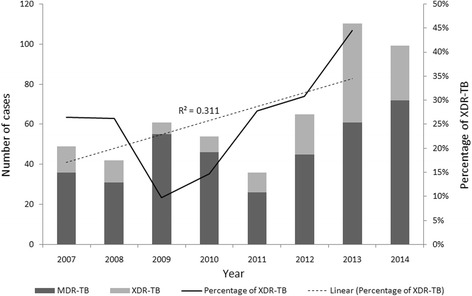
Cases of extensively drug-resistant tuberculosis (XDR-TB) and multidrug-resistant tuberculosis (MDR-TB) in Shandong, China. The percentage of XDR-TB cases in 2015 was 27.3% but was not included in the trend analysis because of incomplete follow-up drug susceptibility data for that year. Chi-Square test for linear trend, 14.097 (*P* < 0.01)

The annual number of XDR-TB cases has fluctuated around a median of 12 cases per year during our study period, and the proportion of XDR TB cases among MDR TB cases has increased from 26.5% in 2008 to 44.5% in 2014 (R^2^ = 0.311; Chi-Square test for trends: χ^2^ = 14.097, *P* < 0.01) (Fig. 2).

Further breakdown into new- and previously treated-TB cases showed that the proportion of XDR TB cases among MDR TB cases has increased in both 2 groups. From 13.3% in 2008 to 41.1% in 2014 in new TB cases (R^2^ = 0.74; Chi-Square test for trends: χ^2^ = 12.894, *P* < 0.001) and from 32.4% in 2008 to 51.4% in 2014 in previously treated TB cases (R^2^ = 0.185; Chi-Square test for trends: χ^2^ = 4.897, *P* = 0.027), respectively.

### Sociodemographic characteristics

The median age of the 144 XDR-TB patients was 42 years (IQR, 27–59 years), and 88 (61.1%) were males.

When compared to MDR-TB and drug-susceptible TB cases, XDR-TB cases were significantly more likely to be in the older age groups (median age, 42 vs. 30 years, *P* < 0.01; 42 vs. 32 years, *P* < 0.01, respectively). Moreover, XDR-TB cases were less likely to be in the student group when compared with drug-susceptible TB cases (5.6% vs. 11.4%, *P* = 0.029; OR 0.46, 95% CI 0.22–0.94). Other sociodemographic characteristics showed no differences between different groups (Table 1).

**Table 1:** Sociodemographic characteristics of extensively drug-resistant tuberculosis (XDR-TB), multidrug-resistant tuberculosis (MDR-TB), and drug-susceptible TB cases, Shandong, China

	NO. (%)			OR (95% CI)	
Characteristics	XDR TB (*n* = 144)	MDR TB (*n* = 372)	Drug-susceptible TB (*n* = 4622)	XDR TB vs. MDR TB	XDR TB vs Drug-susceptible TB
Age, median years (range)	42 (27–59)	30 (24–42)	32 (23–52)	1.04(1.03–1.05)^a^	1.01(1.00–1.02)^a^
Male	88 (61.1)	236 (63.4)	2965 (64.1)	0.91 (0.61–1.35)	0.88 (0.63–1.23)
Occupation					
Farmer	46 (31.9)	113 (30.4)	1169 (25.3)	1.08 (0.71–1.63)	1.39 (0.97–1.98)
Worker	9 (6.2)	28 (7.5)	309 (6.7)	0.82 (0.38–1.78)	0.93 (0.47–1.85)
Student	8 (5.6)	37 (9.9)	527 (11.4)	0.53 (0.24–1.17)	0.46 (0.22–0.94)^b^
Retiree	7 (4.9)	12 (3.2)	249 (5.4)	1.53 (0.59–3.97)	0.90 (0.42–1.94)
Others	74 (51.4)	182 (48.9)	2368 (51.2)	1.10 (0.75–1.62)	1.01 (0.72–1.40)
Alcohol use	17 (11.8)	34 (9.1)	642 (13.9)	1.33 (0.72–2.47)	0.83 (0.50–1.39)
Current Smoker	43 (29.9)	90 (24.2)	1422 (30.8)	1.33 (0.87–2.05)	0.96 (0.67–1.38)

*Abbreviations*: *No.* number, vs versus, *OR* odds ratio, *CI* confidence interval, *MDR* multidrug-resistant, *XDR* extensively drug-resistant

^a^Statistically significant at *P* < 0.001; ^b^Statistically significant at *P* < 0.05

### Clinical and treatment characteristics

Clinical characteristics did not differ between XDR-TB and MDR TB cases (Table 2). However, a previous diagnosis of TB was more than 13 times as likely among XDR-TB cases compared with drug-susceptible TB cases (49.3% vs. 6.5%, *P* < 0.001; OR 13.96, 95% CI 9.87–19.75). And XDR-TB cases were more likely to have disseminated disease (ie. pulmonary and extrapulmonary disease) (16% vs. 9.5%, *P* = 0.01; OR 1.80, 95% CI 1.14–2.85), had a greater chance to have cavity disease (50.7% vs. 35%, *P* < 0.001; OR 1.91, 95% CI 1.37–2.66) and to have bilateral lesions (70.1% vs. 58.3%, *P* = 0.005; OR 1.68, 95% CI 1.17–2.41) in chest radiology compared to drug-susceptible TB. Of note, XDR-TB cases were more likely to have underlying chronic obstructive pulmonary disease (COPD) (4.9% vs. 1.9%, *P* = 0,012; OR 2.63, 95% CI 1.20–5.79) when compared to drug-susceptible TB cases.

**Table 2:** Clinical and treatment characteristics of extensively drug-Resistant tuberculosis (XDR-TB), multidrug-resistant tuberculosis (MDR-TB), and drug-susceptible TB cases, Shandong, China

	NO. (%)			OR (95% CI)	
Characteristics	XDR TB (*n* = 144)	MDR TB (*n* = 372)	Drug-susceptible TB (*n* = 4622)	XDR TB vs MDR TB	XDR TB vs Drug susceptible TB
TB contact^a^	10 (6.9)	42 (11.3)	318 (6.9)	0.59 (0.29–1.20)	1.01 (0.53–1.94)
TB retreatment^b^	71 (49.3)	164 (44.1)	301 (6.5)	1.23 (0.84–1.81)	13.96 (9.87–19.75)^c^
Location of TB disease					
Pulmonary alone	113 (78.5)	313 (84.1)	3939 (85.2)	0.69 (0.42–1.12)	0.63 (0.42–0.95)^d^
Extrapulmonary alone	8 (5.6)	15 (4.0)	242 (5.2)	1 [Reference]	1 [Reference]
Pulmonary and extrapulmonary	23 (16)	44 (11.8)	441 (9.5)	1.42 (0.82–2.45)	1.80 (1.14–2.85)^d^
Chest radiograph result					
Cavity	73 (50.7)	171 (46.0)	1619 (35.0)	1.21 (0.82–1.78)	1.91 (1.37–2.66)^c^
Bilateral disease	101 (70.1)	238 (64.0)	2695 (58.3)	1.32 (0.87–2.00)	1.68 (1.17–2.41)^c^
Comorbidities					
Diabetes	17 (11.8)	35 (9.4)	521 (11.3)	1.29 (0.70–2.38)	1.05 (0.63–1.76)
Hypertension	7 (4.9)	10 (2.7)	269 (5.8)	1.85 (0.69–4.96)	0.83 (0.39–1.79)
Cardio-cerebrovascular disease	6 (4.2)	11 (3.0)	212 (4.6)	1.43 (0.52–3.93)	0.90 (0.40–2.07)
COPD^e^	7 (4.9)	12 (3.2)	88 (1.9)	1.53 (0.59–3.97)	2.63 (1.20–5.79)^d^
Gastric ulcer/Gastrectomy history	7 (4.9)	9 (2.4)	153 (3.3)	2.06 (0.75–5.64)	1.49 (0.69–3.24)
Hepatitis	3 (2.1)	3 (0.8)	67 (1.4)	2.62 (0.52–13.12)	1.45 (0.45–4.66)
Chronic renal failure	1 (0.7)	4 (1.1)	43 (0.9)	0.64 (0.07–5.81)	0.75 (0.10–5.45)
Long-term use of corticosteroids	3 (2.1)	4 (1.1)	65 (1.4)	1.96 (0.43–8.86)	1.49 (0.46–4.80)
Malignancy	2 (1.4)	2 (0.5)	67 (1.4)	2.61 (0.36–18.68)	0.96 (0.23–3.95)
Hypoalbuminemia	9 (6.2)	27 (7.3)	446 (9.6)	0.85 (0.39–1.86)	0.62 (0.32–1.23)
Hepatotoxicity during treatment	18 (12.5)	40 (10.8)	534 (11.6)	1.19 (0.66–2.15)	1.09 (0.66–1.81)
DOT					
Complete DOT^f^	92 (63.9)	308 (82.8)	4450 (96.3)	1 [Reference]	1 [Reference]
Partial DOT	52 (36.1)	64 (17.2)	172 (3.7)	2.72 (1.76–4.20)^c^	14.62 (10.08–21.23)^c^
Hospital LOS	78 (45–132)	70 (41–103)	46 (34–65)	*P* = 0.025	*P* < 0.001

*Abbreviations*: *No.* number, vs versus, *OR* odds ratio, *CI* confidence interval, *MDR* multidrug-resistant, *XDR* extensively drug-resistant, *COPD* chronic obstructive pulmonary disease; *DOT* directly observed therapy, *LOS* length of stay

^a^TB contact was defined as family members, schoolmates or colleagues with TB

^b^TB retreatment means that the case has received TB treatment before the current TB episode for one month or more [20]

^c^Statistically significant at *P* < 0.01

^d^Statistically significant at *P* < 0.05

^e^COPD was diagnosed according to the guidelines of Global Initiative for Chronic Obstructive Lung Disease (GOLD). Defined by a post-bronchodilator pulmonary function (forced expired volume in one second (FEV1)/forced vital capacity (FVC) ratio) of less than 70%, with prescription of a combination of various bronchodilators and anticholinergic agents. In our study, all COPD patients had been diagnosed before TB diagnosis

^f^Complete DOT refers to TB treatment under direct observation of health workers from hospitals/communities and trained family members; Partial DOT refers to DOT combined with some self-administered therapy

For treatment characteristics, XDR-TB cases were more likely to have received treatment under partial rather than total directly observed therapy when compared with MDR TB and drug-susceptible TB cases (OR 2.72, 95% CI 1.76–4.20 vs. MDR TB; OR 14.62, 95% CI 10.08–21.23 vs. drug-susceptible TB), and to stay longer in the hospital (median LOS, 78 vs. 70 days, *P* = 0.025 when compared to MDR TB cases; 78 vs. 46 days, *P* < 0.001 when compare to drug-susceptible TB cases).

### Treatment outcomes

XDR TB patients had significantly worse outcomes than MDR TB and drug susceptible TB cases. We found that patients with XDR TB had a relative risk of 2.33 to die (95% CI, 1.05–5.16; *P* = 0.033) and of 3.19 to have treatment failure (95% CI, 2.11–4.82; *P* < 0.001), compared with patients with MDR TB. And it is even more prominent when compared with drug susceptible TB cases, with a relative risk of 4.24 to die (95% CI, 2.27–7.92; *P* < 0.001) and of 9.96 to have treatment failure (95% CI, 7.05–14.06; *P* < 0.001) (Table 3).

**Table 3:** Treatment outcomes of patients with XDR-TB, MDR TB and drug susceptible TB

	NO. (%)			OR (95% CI)	
Treatment outcomes	XDR TB (*n* = 144)	MDR TB (*n* = 372)	Drug-susceptible TB (*n* = 4622)	XDR TB vs MDR TB	XDR TB vs Drug susceptible TB
Treatment Success					
Cure	8 (5.6)	51 (13.7)	1719 (37.2)	0.37(0.17–0.80)^a^	0.10(0.05–0.20)^b^
Treatment completion	13 (9)	115 (30.9)	1248(27.0)	0.22(0.12–0.41)^b^	0.27(0.15–0.48)^b^
Poor Treatment Outcome					
Death	12 (8.3)	14 (3.8)	97(2.1)	2.33(1.05–5.16)^a^	4.24(2.27–7.92)^b^
Failure	66 (45.8)	78 (21.0)	362(7.8)	3.19(2.11–4.82)^b^	9.96(7.05–14.06)^b^
Default	6 (4.2)	12 (3.2)	91(2.0)	1.30(0.48–3.54)	2.17(0.93–5.03)
Transfer out/unknown/missing	39 (27.1)	102 (27.4)	1105(23.9)	1 [Reference]	1 [Reference]

^a^Statistically significant at *P* < 0.05

^b^Statistically significant at *P* < 0.001

### Survival analysis

Participants were followed up for a range of 12.3 to 109.4 months (median 45.5; IQR, 29.1–75.9), starting from initiation of treatment to the end of follow-up in September 30, 2016 or to the date of death.

**Fig. 3:**
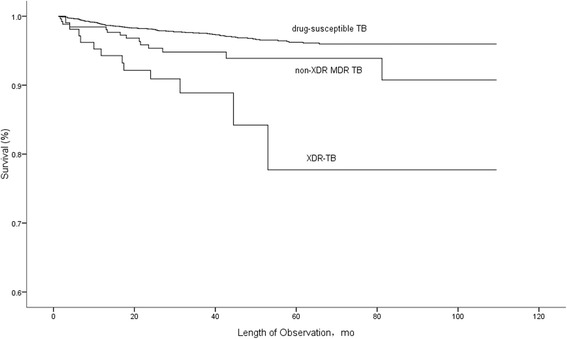
Survival among XDR-TB, MDR-TB and drug-susceptible TB cases, Shandong, China. Among patients included in the survival analysis, there were 12 deaths (11.4%) in the XDR-TB group, 14 deaths (5.4%) in the MDR TB group, and 97 deaths (2.8%) in the drug-susceptible TB group. Survival curves were compared using the log-rank test (*P* = 0.016 for XDR-TB vs. MDR TB and *P* < 0.01 for XDR-TB vs. drug-susceptible TB)

Figure 3 shows Kaplan-Meier estimates of survival based on the number of deaths from any cause among patients with XDR-TB, MDR TB and drug-susceptible TB. Survival was statistically worse among XDR-TB cases compared with both MDR TB cases (χ^2^ = 5.806, *P* = 0.016) and drug-susceptible TB cases (χ^2^ = 35.603, *P* < 0.001) using the log-rank test.

Within one year, 6 XDR-TB cases (5.7%) had died during treatment compared with 4 MDR TB cases (1.6%; *P* = 0.038) and 41 drug-susceptible TB cases (1.2%; *P* = 0.002).

## Discussion

It is a large population and long term-based retrospective study that provides the first comprehensive assessment of XDR-TB in China and represent 8 years of the surveillance data almost from the first terming of XDR-TB in 2006.

The emergence of XDR-TB globally has raised awareness and concern about incurable forms of TB. Between January 2008 and December 2015, a total of 144 XDR-TB cases were identified, accounting for 27.9% of MDR-TB cases in our sample population, which is way above the average level (9%) globally in 2014 [15]. By contrast, a rate of 6.3%–12.8% has been documented in most of the previous studies from different regions of China, revealing the high prevalence of XDR-TB in our setting [16–18, 23, 24]. However, the rate is quite similar to the 23.59% from 2010 nationwide population-based survey in China [25]. The high prevalence of XDR-TB could be explained by the fact that our study was a provincial TB hospital based surveillance study, with a relatively high proportion of intractable patients. Second, in previous studies, not all of the MDR-TB cases were XDR-TB evaluable and there were less second line drugs included in the DST panel, which may have led to selection bias and underestimation of the XDR TB prevalence.

It is also noteworthy that the proportion of XDR-TB cases among MDR-TB cases increased from 26.5% in 2008 to 44.5% in 2014, increasing at a yearly rate of 2.9% throughout the study period. Separate analysis of new- and previously treated- TB cases showed that the increase of this proportion is due to the increase of XDR-TB proportion in both 2 groups. There have been few reports about the longitudinal changes of XDR-TB in China, the increase of XDR-TB in both new- and previously treated- TB cases in our study is a wake-up call for the inefficiency of existing TB control policies towards XDR-TB.

In our study, the outcomes of XDR-TB cases were poorer than MDR-TB cases just like other studies. The treatment success rates were 14.6% for XDR-TB cases and 44.6% for MDR-TB cases, respectively. And the 14.6% success rate of XDR-TB treatment was much lower than 29.2–60.4% rates in previous studies [11, 26–29]. One of the possible reasons is the relatively high loss-to-follow-up rates in our study. Second, treatment of XDR-TB cost a lot and free second-line TB drugs are not routinely available in China, leading to intermittent or irregular treatment. Third, DST are not routinely performed in many unspecialized hospitals, XDR-TB cannot be found and treated as early as possible, which has a great impact on treatment effectiveness.

Due to the absence of HIV co-infection, mortalities of TB cases in our study are much lower than previous studies with a relatively high prevalence of HIV infection [3, 8, 13, 30]. But still, the death rates were significantly higher among XDR-TB cases compared with both MDR TB cases and drug-susceptible TB cases in our study, which was in consistent with previous studies [3, 13].

XDR TB and drug-sensitive TB patients have very distinct differences in clinical characteristics. XDR TB cases are more likely to have cavity, bilateral and extrapulmonary disease than drug-sensitive TB cases. It could be closely related to their underlying duration of disease. Due to the belated drug susceptibility testing and their belated results, XDR- and MDR-TB patients often are inappropriately put on first-line therapy which they inevitably fail. Significant time passes before these patients are recognized as having MDR or XDR TB allowing for disease progression. On the other hand, it also have important implications that XDR TB are more likely to be infectious. Cavity is verified to be a symbol of TB infectivity, and a previous study has shown that XDR TB cases have prolonged infectious period of approximately 6 months, underscores the urgent need for improving the prevention, diagnosis, and treatment of XDR-TB [13].

It is not a total surprise that we found XDR-TB cases were more likely to be in the older age groups and to have underlying COPD. COPD is a major public health problem in China, accounting for 8.2% individuals aged >40 years [31], they are usually malnourished or have compromised immunities, potentially increase the chance of active TB infection [32, 33]. In addition, a substantial proportion of COPD patients experience reiterative hospitalization and antibiotic treatment, selection and nosocomial transmission of resistant strains are thus inevitable in these individuals [34–38]. It has been documented that COPD was an independent risk factor associated with fluoroquinolone resistant tuberculosis in a previous study [39]. In the present study, 4.9% of XDR-TB cases were complicated with COPD, which is a relatively high proportion calling for our attention. TB patients with COPD should be noticed and DST for second line drugs should be performed at the very beginning of the disease to identify possible XDR-TB cases, moreover, treatment strategies and optimal duration for XDR-TB patients with underlying COPD remains to be determined.

Limitations of our findings are due to the retrospective study design. Detailed TB treatment histories, genotyping and molecular typing data are not included in case reporting. Though we founded that 45.1% of XDR-TB cases in our study were from primary transmission, the relative importance of primary XDR-TB transmission vs. additional drug resistance during treatment is still undeterminable. And possible exogenous reinfection during hospitalization cannot be ruled out.

## Conclusions

The present study has important implications for TB control efforts in China. The sizable proportion of XDR-TB cases among MDR-TB cases and its increasing trend in both new- and previously treated- TB cases as well as the poor treatment outcomes sounded the alarm about the inefficiency of existing TB control policies towards XDR-TB. Delayed drug susceptibility testing, suboptimal prescriptions and inadequate infection control precautions may have contributed to the development of XDR TB strains in our study population. Lessons learned from MDR-TB in the 1990s should be applied: efficient infection control, prompt diagnosis, early drug susceptibility testing, adequate TB treatment, and consistent use of directly observed therapy must be in place to prevent further emergence and transmission of XDR-TB in places with similar settings.

## Abbreviations

CI: Confidence interval
COPD: Chronic obstructive pulmonary disease
DOT: Directly observed therapy
DR-TB: Drug-resistant tuberculosis
DST: Drug susceptibility testing
FEV1: Forced expired volume in 1 s
FVC: Forced vital capacity
GOLD: Global Initiative for Chronic Obstructive Lung Disease
HIV: Human immunodeficiency virus
IQR: Inter quartile range
IUATLD: The International Union Against Tuberculosis and Lung Disease
LOS: Length of stay
MDR-TB: Multidrug-resistant tuberculosis
NTM: Non-tuberculous mycobacteria
OR: Odds ratio
SPCH: Shandong Provincial Chest Hospital
TB: Tuberculosis
WHO: World Health Organization
XDR-TB: Extensively drug-resistant tuberculosis
